# Direct interaction between p53 and Tid1 proteins affects p53 mitochondrial localization and apoptosis

**DOI:** 10.18632/oncotarget.174

**Published:** 2010-09-30

**Authors:** Diane L.N. Trinh, Adam N. Elwi, Sung-Woo Kim

**Affiliations:** ^1^ Department of Biochemistry & Molecular Biology, University of Calgary, Calgary, Alberta, T2N 4N1, Canada; ^2^ Southern Alberta Cancer Research Institute, Calgary, Alberta, T2N 4N1, Canada; ^3^ Clark H Smith Brain Tumour Centre, Calgary, Alberta, T2N 4N1, Canada

**Keywords:** apoptosis, mitochondria, p53, Tid1

## Abstract

The p53 tumor suppressor induces apoptosis in response to genotoxic and environmental stresses. Separately from its functions as a transcription factor, it is also capable to be translocated to the mitochondria and plays a critical role in transcription-independent mitochondrial apoptosis. We previously demonstrated that Tid1 interacts with p53, resulting in mitochondrial translocation of the complex and induction of intrinsic apoptosis [1]; however, the mechanism how they interact has been unknown. In this study, far western analyses demonstrated that Tid1 directly interacted with p53. Using domain deletion mutant constructs, we determined that DnaJ domain of Tid1 was necessary for the interaction, while either N- or C-terminal domains of p53 were sufficient for the interaction. In breast cancer cells, depletion of Tid1 by short hairpin RNA (shRNA) led to absence of p53 accumulation at mitochondria and resistance to apoptosis under hypoxic or genotoxic stresses. Our studies imply that Tid1 could be important in the potential combination chemotherapies of p53-related cancers.

## INTRODUCTION

p53 has been known for many years to play a large role as a transcription factor in the nucleus of cells in regulation of the transcription of over 150 target genes involved in growth, proliferation, senescence, apoptosis, and invasion [2,3,4,5,6]. The use of a temperature sensitive p53Val135 mutant in combination with inhibitors of transcription and translation established that p53 also has a transcription-independent function in apoptosis [7,8]. In 2000, it was reported that a small fraction of p53 induced by cellular stress would quickly translocate to the mitochondria as a first wave rapid response only during a p53-dependent apoptotic program [2,7,9,10,11]. This translocation was a general phenomenon to wildtype p53 harboring malignant and non-malignant cells and occurred prior to mitochondrial membrane potential changes, the apoptotic release of cytochrome *c*, and activation of the caspase cascade [9,10]. The direct targeting of p53 to the mitochondria by fusion of the mitochondrial import leader peptide of human ornithine transcarbamylase, or the transmembrane domains of anti-apoptotic proteins Bcl-xL or Bcl2 to p53 was sufficient to launch an apoptotic program [7,9,12].

Tid1 is a type I member of the DnaJ family of molecular chaperones as it possesses not only the DnaJ domain, but also Cys-rich and G/F rich domains [13,14,15,16,17]. This DnaJ domain, in particular the HPD motif, has been shown to be important for the interaction with the heat shock 70 [Hsp70] family of proteins to allocate for substrate specificity as well as ATPase activity [15,17,18,19]. The interaction between Tid1 and Hsp70 has been demonstrated in a number of studies, and also shown to be significant for the interaction with other proteins such as the viral Tax protein and the ErbB-2 receptor tyrosine kinase [20,21]. Mutations in the DnaJ domain affected the ErbB-2 interaction that lead to reductions in Tid1-mediated apoptosis [21]. The N-terminal domain of Tid1 has also been relatively well characterized on the basis of the presence of consensus sequences and by mutational studies. The consensus mitochondrial targeting (MAARCS) and cleavage sequences (LRPGV) allow for the translocation of Tid1 to the mitochondria where metalloproteases cleave the precursor to form the mature protein [22,23,24]. Tid1 mutants of either isoform, which lack the N-terminal targeting sequence, did not co-localize with the mitochondria, but rather were dispersed throughout the cytosol [22]. Tid1 was previously shown to interact with p53 *in vivo*, particularly in the mitochondrial fractions, leading to apoptosis [1]; however, little is known of the nature and significance that protein domains of Tid1 and p53 play in their interaction. Here we show that Tid1 directly interacts with p53 mostly likely through its DnaJ domain and its depletion induces resistance to stresses by inhibiting the p53 localization to the mitochondria.

## RESULTS

### Cellular localization of Tid1 full length and its deletion mutants

**Figure 1:**
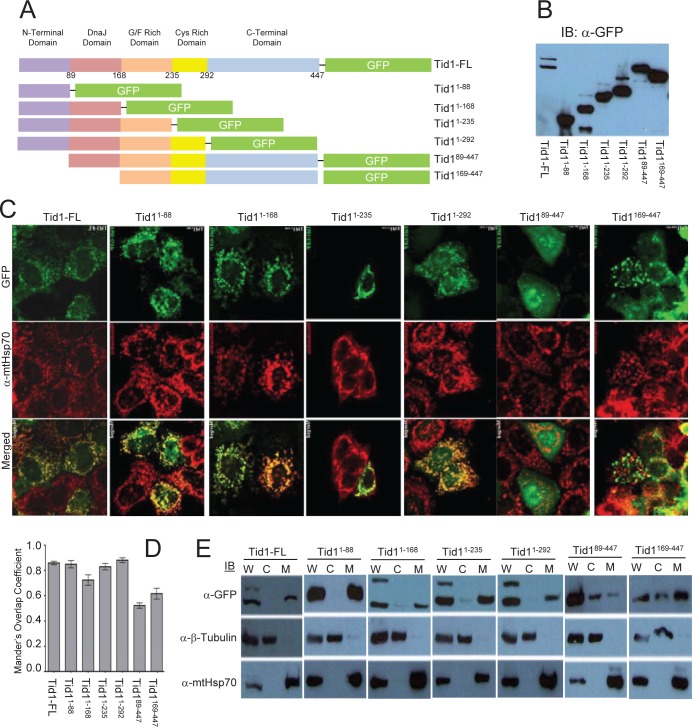
Cellular localization of Tid1 full length and its deletion mutants MCF-7 cells were transfected with Tid1 constructs to overexpress Tid1 full length or its domain deletion mutants (A) Schematic diagram of Tid1-EGFP full length or the domain deletion mutants (B) Immunoblotting analysis for Tid1 and its mutants. (C) Tid1-EGFP transfected cells labeled with anti-mtHsp70 were visualized by confocal microscope. (D) Mander's Overlap Co-efficient between the various Tid1 constructs and mtHsp70 as determined by Image J Intensity Correlation Analysis Software, with 1 being a high colocalization and 0 being low (n=4). Results are representative of a minimum of two independent experiments. (E) Cells were subjected to biochemical fractionation (W = whole cell extracts; C = cytosolic fraction; and M = mitochondrial fraction) followed by immunoblotting. Purity of the fractions was determined by immunoblotting with anti-mtHsp70 (mitochondrial) and anti-β-tubulin (cytosolic) antibodies.

We first tested whether the loss of certain domains of Tid1 had any effect on the mitochondrial localization. The pEGFP-Tid1 wildtype and mutants constructs (Fig. 1A) were transfected into the human breast cancer MCF-7 cell line and immunoblotting was performed using anti-GFP antibody to checked the molecular weight of the generated fusion proteins and their proper processing (Fig. 1B). For detection of the mitochondria, immunostaining and confocal microscopy were performed with the anti-mtHsp70 antibodies. Similar to endogenous Tid1, the full length form of Tid1-GFP was found to mainly co-localize with the mtHsp70 staining, indicating that this fusion protein also translocated to the mitochondria (Fig. 1C). Consecutive truncations of the C-terminal end of Tid1 did not appear to affect the mitochondrial localization of the Tid1 mutants (Fig. 1C). As expected, the loss of the N-terminal domain (amino acid residues 1-88) resulted in a dramatic reduction of co-localization of the Tid1 mutants with mtHsp70 (Fig. 1C). We quantified the degree of co-localization of our exogenous Tid1 and mtHsp70 (Fig. 1D) and found that Tid1-FL and the N-terminal containing mutants all had greater than 0.70 correlation coefficients indicating relatively high co-localization with mtHsp70, whereas the N-terminal deletion constructs had lower co-localization correlation coefficients (approximately 0.50). Biochemical fractionations using a cell compartment fractionation kit, confirmed our immunofluorescent findings that full length Tid1 and the mutants possessing the N-terminal domain, were found primarily in the mitochondrial fraction of the cell (Fig. 1E). Note that there are two bands present in the whole cell extracts of the Tid1_1-168_, Tid1_1-235_, and Tid1_1-292_ mutants as these mutants are processed in the mitochondria in the same manner as endogenous full length Tid1. Hence, our biochemical and immunofluorescent data confirm previous notions of the importance of the N-terminal domain in the mitochondrial localization of Tid1.

### Cellular localization of p53 full length and its deletion mutants

**Figure 2:**
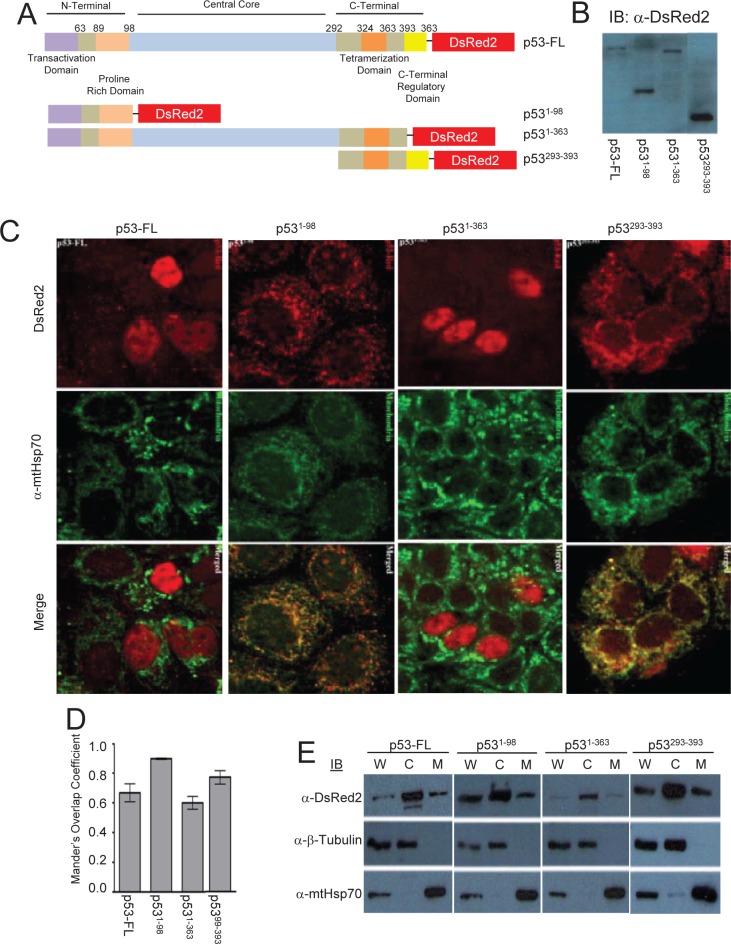
Cellular localization of p53 full length and its deletion mutants MCF-7 cells were transfected with p53 constructs to overexpress p53 full length or its domain deletion mutants (A) Schematic diagram of p53-DsRed2 full length or domain deletion mutants (B) Immunoblotting analysis for p53 and its mutants. (C) p53-DsRed2 transfected cells labeled with anti-mtHsp70 were visualized by confocal microscope. (D) Mander's Overlap Co-efficient between the various p53 constructs and mtHsp70 as determined as described in Fig. 1 D. (E) Cellular fraction were obtained as described in Fig. 1 E.

Parallel immunofluorescent experiments to the Tid1 mutants were performed with the pDsRed2-p53 wildtype and mutants (Fig. 2A). We transfected these constructs into MCF-7 cells, and through immunoblotting with anti-DsRed2 antibody, checked the molecular weight of the generated fusion proteins (Fig. 2B). A majority of exogenous full length p53 and p531-363 was largely found in the nucleus of cells although some staining in the cytoplasmic portions of the cells was detected (Fig. 2C). An average co-localization correlation coefficient of 0.70 was quantitated for full length p53 and mtHsp70 (Fig. 2D), indicating some colocalization. Loss of the C-terminal region of p53 as with p531-98, resulted in the nuclear exclusion of p53, due to the absence of the nuclear localization sequence (NLS), and relatively high mtHsp70 colocalization with a correlation coefficient of 0.90. p531-363 was primarily nuclear with a correlation coefficient of 0.60 (Fig. 2D). The C-terminal domain mutant, p53293-393 has a large proportion found in the insoluble cytoskeletal fraction (data not shown). These findings agree with those from Wadhwa *et al.* [25], that the NLS is required for the nuclear translocation of p53; however, the existence of a cytoplasmic sequestration domain in the C-terminal region of p53 can lend to nuclear exclusion.

### The DnaJ domain of Tid1 is required for the interaction to both the N- and C-terminal domains of p53.

**Figure 3:**
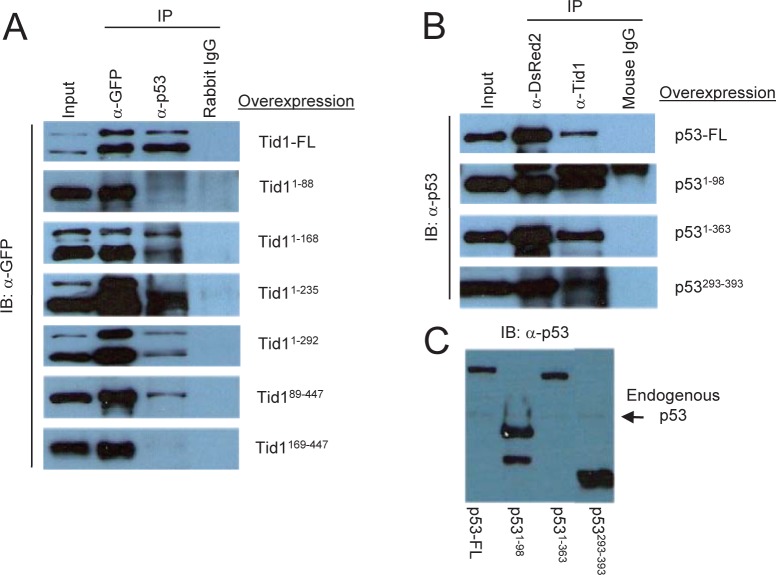
DnaJ domain of Tid1 and both N- and C-terminal domain of p53 are important for their interaction. (A) Wildtype or the different mutant Tid1-EGFP constructs were introduced into MCF-7 (p53^wt^) cells by Lipofectamine2000 transfection. Cells were lysed, immunoprecipitated with the indicated antibodies and immunoblotted with anti-GFP antibodies. Rabbit IgG was used as the negative control (B) Wildtype or the different mutant p53-DsRed2 constructs were introduced into MCF-7 (p53^wt^) cells. Cells were lysed, immunoprecipitated with the indicated antibodies and immunoblotted with anti-p53 antibodies. Mouse IgG was used as the negative control. (C) Immunoblotting analysis of p53 and its mutants with anti-p53 antibodies.

Next, we determined which domains of both Tid1 and p53 were required for their interaction. The various pEGFP-Tid1 constructs were transfected into MCF-7 cells to exogenously produce these Tid1 mutant proteins. Protein lysates were then utilized for immunoprecipitation with a rabbit polyclonal anti-GFP antibody, polyclonal anti-p53 antibody, or normal rabbit IgG and Protein A/G agarose beads. These samples were then immunoblotted with a monoclonal anti-GFP antibody. A consecutive loss of the non-homologous C-terminal, Cys-rich, and G/F-rich domains starting from the C-terminal end of Tid1 did not appear to interfere with the interaction between Tid1 and endogenous p53. Although the Tid1^89-447^ mutant mislocalized in the cell (Fig. 1C), we still detected a relatively strong interaction between this mutant and p53 (Fig. 3A). The additional loss of the DnaJ domain from the N-terminal end of Tid1 resulted in the abrogation of the Tid1/p53 interaction (Fig. 3A). Thus, consecutive domain deletions from either end of the Tid1 protein have exemplified the importance of the DnaJ domain for the formation of this protein complex.

To determine the region of interaction on p53, pDsRed2-p53 constructs were transfected in MCF-7 cells and the protein lysates were used for immunoprecipitation with the monoclonal DsRed2 antibody, monoclonal anti-Tid1 antibody, or normal mouse IgG and Protein A/G-agarose (Fig. 3B). The immunoprecipitates were then immunoblotted with anti-p53 antibody (FL393) (Fig. 3C). All exogenous p53 wildtype and mutants were detected (Fig. 3B). Though it was not clear if the transactivation domain is required for the interaction, these data showed that either the N-terminal domain or C-terminal region of p53 was sufficient for p53 to maintain an interaction with Tid1.

### Tid1 directly interacts with p53

**Figure 4:**
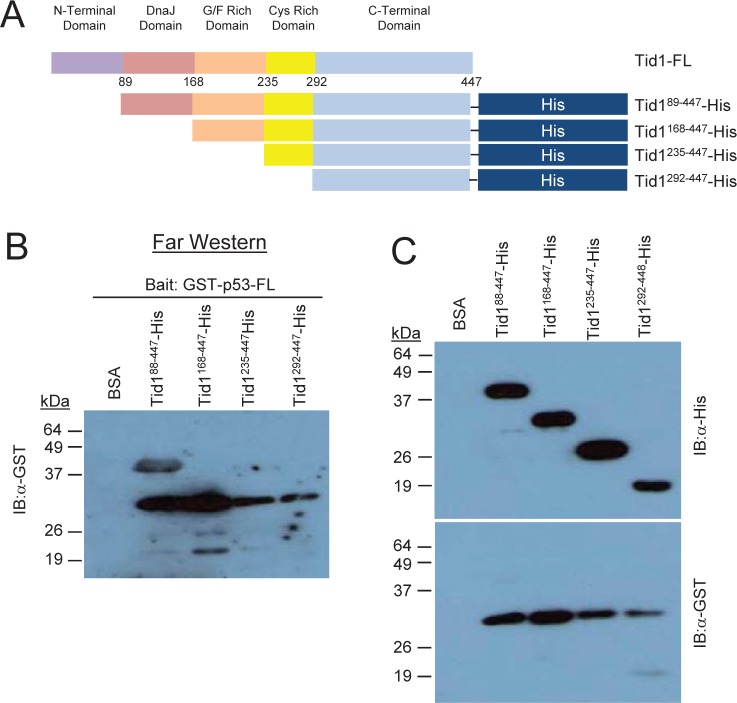
Tid1 directly interacts with p53 Schematic diagram of His-tagged Tid1 full length or domain deletion mutants (B) Purified His-tagged Tid1 mutant proteins were resolved on nitrocellulose membranes, incubated with GST tagged p53 protein and immunoblotted with anti-GST antibodies. (C) The membrane loaded with His tagged Tid1 mutant proteins were immunoblotted with anti-His or –GST antibodies.

To determine if these proteins directly interacted or whether another protein is involved in the complex, we used the *in vitro* Far Western approach. As the consecutive deletion of the C-terminal, Cys-rich and G/F-rich domains from the C-terminal end of Tid1 did not appear to interfere with the interaction between Tid1 and endogenous p53 (Fig. 3A), we focused on the domains deleted from the N-terminal. His-tagged Tid1 mutant proteins (Fig. 4A) were purified. Unfortunately, we were unable to generate purified proteins for full length Tid1 or any Tid1 mutants possessing the N-terminal domain, in spite of numerous efforts to optimize the protein expression. Despite this, we were able to obtain the N-terminal-lacking domain deletion mutant Tid199-447, which was shown through our co-immunoprecipitation to interact with p53. The purified His-tagged proteins were resolved on the membrane to use as prey proteins. The membrane was washed and incubated with the purified N-terminal GST-tagged p53 proteins as bait, followed by an anti-GST antibody to detect the presence of bait binding. While Tid199-447 bound GST-p53, no binding to GST-p53 to the Tid1235-447 and Tid1229-447 mutants was detected (Fig. 4B). Unfortunately, we were unable to answer whether Tid1168-447 bound p53-GST directly (Fig. 4B) as anti-GST antibody generated a non-specific band at the band size as the mutant (Fig. 4C bottom panel). These data imply that Tid1 can bind p53 directly and in this interaction, the Cys-rich and C-terminal domains of Tid1 are not required.

### Suppression of Tid1 leads to absence of p53 at mitochondria and apoptosis following cellular stresses

**Figure 5:**
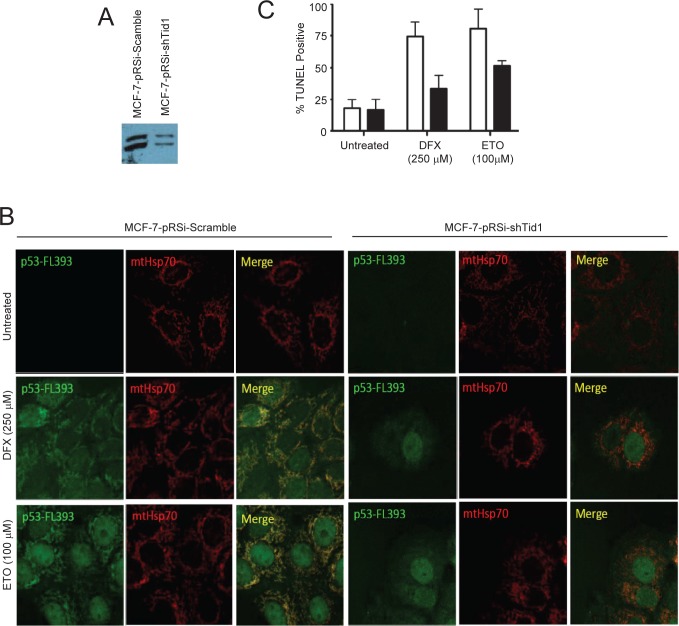
Suppression of Tid1 leads to loss of p53 at mitochondria and resistance to apoptosis following cellular stresses. MCF-7 cells stably expressing non-silencing shRNA (shScram) or shRNA directed against Tid1 (shTid1) were incubated in the presence or absence of 250 μM DFX or 100 μM for ETO 6 hr. (A) Tid1 expression in MCF-7 cells stably expressing shScram or shTid1. (B) Cells labeled with anti-p53 antibodies (green) and anti-mtHsp70 antibodies (red) were visualized by confocal microscopy. (C) Apoptotic cells were detected by *in situ* TUNEL assay.

To finally determine if Tid1 has a functional role in p53 localization and apoptosis under various cellular stresses, we generated MCF-7 cell lines stably suppressing Tid1 (Fig. 5A) by shRNA. Cells in which Tid1 was knocked down (MCF7-shTid1) had significantly less mitochondrial p53 (Fig. 5B) and lower levels of apoptosis (Fig. 5C) compared to that seen in the control under treatment of either desferroxamine mesylate (DFX), a hypoxia mimetic, or etoposide (ETO), a genotoxic stress. This corresponded to previous results from our laboratory using DFX but the effects seen here are at higher doses of DFX than we previously used [1]. These results further extend our previous findings [1] that depletion of Tid1 expression inhibits hypoxia or genotoxic stress-induced p53 mitochondrial localization and apoptosis. Our data suggest that the binding of Tid1 to p53 may affect p53's translocation to mitochondria.

## DISCUSSION

Protein interactions with the Tid1 tumor suppressor have demonstrated the importance of Tid1 in a variety of signaling pathways that control cellular growth, proliferation, differentiation, senescence, and apoptosis. These interactions, like many others, have the requirement of proper cellular localization of the proteins in the cell. Mislocalization, or sequestration of a protein in different cellular compartments, can alter their functional interactions and activities. Transcription factors, for instance, are required to localize to the nucleus to bind to DNA for the induction of transcriptional activity of target genes. p53 is a well known transcription factor that regulates the expression of over 150 target genes involved in cell cycle progression, cellular senescence, and apoptosis [2,3,4,5,6]. The loss or mutation of its NLS sequences results in the abrogation of nuclear targeting and thus its function as a transcription factor. In addition to this, it was shown that p53 interacts with mtHsp70 near its C-terminal end, which results in the cytoplasmic sequestration of p53 leading to the inactivation of its nuclear transactivation function [25,26]. This clearly demonstrates the significance of cellular localization of p53 protein function.

Although cytoplasmic sequestration compromises the transcriptional function of p53, studies have also shown it to have a transcription-independent function in apoptosis. This function is dependent on its localization to the mitochondria. We believe that an interaction with Tid1 may play a role in this translocation as we have previously shown that Tid1 and p53 interact with one another as well as co-localize with each other and with the mitochondria under conditions of cellular stress [1].

Here, we wanted to test the effects of the loss of various domains of both Tid1 and p53 on their cellular localization and their ability to interact with one another. We found that in the presence of the N-terminal domain, and thus the presence of the mitochondrial targeting and cleavage sequences, the deletion of the other domains from Tid1 did not appear to alter its primarily mitochondrial localization. The loss of this domain, on the other hand, resulted in the mislocalization and potential aggregation of the Tid1 mutants in the cell. Like with many other mitochondrial targeted proteins, we have confirmed the significance of the N-terminal domain in the cellular localization of this protein.

As many mitochondrial precursor proteins are encoded by nuclear genes, they are synthesized in the cytosol prior to transport to the mitochondria [27]. Cytosolic factors and chaperonic proteins, such as members of the heat shock 70 family are likely involved in the proper folding and prevention of aggregation of these precursor proteins [27]. In the absence of this N-terminal pre-sequence, not only is targeting to the mitochondria lost, but misfolding of the protein appears to occur. Although it is beyond the scope of this study, there are continuing efforts to determine the exact role of the N-terminal domain in protein mislocalization and aggregation and its link to cancer.

With our p53 domain deletion constructs, our immunofluorescent and biochemical data was, in large part, in agreement with the previous findings from Kaul *et al.*, [26]. They utilized N-terminal YFP-tagged p53 proteins to determine the localization through an immunofluorescent approach. This indicates that the large, approximately 26kDa fluorescent tag does not appear to interfere with protein localization. We found that p53-FL was localized to all fractions of the cell, although it was primarily visualized to be nuclear through immunofluorescence. This is in slight disagreement with Kaul *et al.* [26] as they concluded that p53-FL is exclusively nuclear. This observed difference may be the result of the use of an overexpression system and thus mislocalization of exogenous protein can occur, depending on the degree of overexpression.

The absence of the NLS resulted in exclusively cytoplasmic protein, but the presence of the NLS with the presence of the cytoplasmic sequestration region (amino acid residues 323-337) and the mortalin binding domain (amino acid residues 312-352), resulted in at least some cytoplasmic localization of the p53 mutants [26]. Of note, the loss of the N-terminal domain shifted the primary localization of the proteins from the nuclear fraction to the cytoplasmic fraction. This indicates a possible role of the N-terminal domain in addition to the C-terminal domain in cytoplasmic sequestration. Our co-immunoprecipitation data indicates that the N-terminal domain is sufficient for the interaction with Tid1 and the loss of this domain abrogated the formation of the complex. This suggests that Tid1 may be involved with mtHsp70 in the translocation and cytoplasmic sequestration of p53.

With our Tid1 mutants, we demonstrated the significance of the DnaJ domain for interaction with endogenous p53. This indicates an involvement of the heat shock 70 family of molecular chaperones, particularly of mortalin/mtHsp70 which has already been shown to interact with both Tid1 and p53. As DnaJ/DnaK or Hsp40/Hsp70 interactions are important for proper protein folding, translocation, degradation, and the assembly/disassembly of complexes [16-18], further studies are required to determine the exact role of mtHsp70 in the Tid1/p53 interaction and perhaps its role in the protein aggregation seen in the absence of the N-terminal domain.

Together, our data implicates a multi-step interaction between Tid1 and p53 which involves multiple domains on both proteins. Studies have already demonstrated the formation of Hsp70/Hsp40/p53 complexes in cells providing further evidence supporting our proposed model. Sugito *et al.* [28], provided the first evidence for the existence of an Hsp70/mtp53//Hsp40 ternary complex and suggested the existence of an Hsp70 (DnaK)/Hsp40 (DnaJ) chaperone system that is involved in the repair of unfolded proteins or mutant p53. The cytosolic form of Hsp70, Hsc70, was also found to interact with both wildtype and mutant p53, but only in the presence of Hsp40 and ATP [29]. The interaction between Hsp40 and p53 was shown to be relatively strong, yet the interaction between p53 and Hsp70 was weak and transient, suggesting that Hsp40 may be the driving factor for the formation of stable ternary protein complexes [29].

## MATERIALS AND METHODS

### Generation of Domain Deletion Mutant Constructs

In addition to the full length form of Tid1, we generated a total of six domain deletion constructs with consecutive domains deleted from either end of the protein (Fig 1A). These constructs were cloned into pEGFP-N. Similar to the Tid1 domain deletion constructs, p53wt and its mutants were cloned into the pDsRed2-N plasmid (Fig. 2A). For the study of direct interaction, the Tid1^wt^ and its mutants constructs were cloned into the pET-22b(+) bacterial expression vector that contains C-terminal His-tagged fusion proteins. The p53wt construct was cloned into the pGEX-4T3 bacterial expression vector that contains cleavable N-terminal GST-tagged fusion proteins.

### Cell culture and transfection

The human breast cancer cell line MCF-7 was grown in Dulbelcco's Modified Eagle Medium (DMEM, Invitrogen) supplemented with 10% fetal bovine serum (FBS, Invitrogen) and antibiotics-antimycotics (Invitrogen). Cells were tranfected with 5-10μg of plasmid DNA in 10cm plate using Lipofectamine2000 (Invitrogen) according to the manufacturer's instructions

### Subcellular Fractionation, Immunoprecipitation, Immunoblotting and Confocal Microscopy

These methods were performed as described in our previous study [1]. Anti-p53 (FL-393), anti-Tid1 (RS-11), anti-β-tubulin (H-235), anti-GFP (B2) or anti-DsRed2 (Santa Cruz Biotechnology) and anti-mtHsp70 (MA3-028, Transduction Labs, Oxford, UK) antibodies were used for immunoprecipitation or immunoblotting. For confocal microscopy, stained cells were examined using a Zeiss LSM 510 Laser Scanning Confocal Microscope.

### Protein Amplification and Purification

Both the pET-22b-Tid1 and pGEX-4T3-p53 bacterial constructs were transformed into BL21 cells chemically competent cells for the expression of our genes of interest. These were then purified using the Ni-NTA agarose system (for His-tagged constructs) or Glutathione-Sepharose beads (for GST tagged constructs), respectively. The purity of all our protein was >90% as determined by SDS-PAGE Coomassie Brilliant Blue staining (data not shown).

### Far westerns

1 μg of purified His-tagged Tid1 mutants were run on SDS-PAGE and transferred to nitrocellulose membrane. The membranes were then denatured and re-natured using AC buffer containing 6M Guanidine-HCl (Sigma) for 30 minutes at room temperature, 3M Guanidine-HCl for 30 minutes at room temperature, 1M Guanidine-HCl for 30 minutes at room temperature, 0.1M Guanidine-HCl for 30 minutes at 4°C, and no Guanidine-HCl overnight at 4°C. Membranes were washed 3 times with PBST, blocked for 1 hour in 5% skim milk in PBST, and incubated with 5μg of bait protein in binding buffer (100mM NaCl, 20mM Tris [pH 7.^6^], 0.5mM EDTA, 10% glycerol, 0.1% Tween-20, 2% skim milk powder and 1mM Dithiothreitol [DTT, Sigma]) overnight at 4°C. Bound bait protein was detected by a primary antibody in a method similar to standard Immunoblotting described. Membranes were stripped and reprobed using standard Immunoblotting to determine the position of the prey proteins on the membrane. Anti-His (H-15) and anti-GST (1-109) (Santacruz Biotechnology) were used for detection of the tagged constructs.

### Tid1 Knock down and stable cell lines

MCF7 cells were infected with retrovirus amplified from pRetroSuper constructs containing short hairpin RNA (shRNA) directed against Tid1 (shTid1, 5'-CAGCTACGGCTACGGAGAC-3') or non-silencing shRNA (shSCRAM, 5'-AATTCTCCGAACGTGTCACGT-3') and selected for two weeks in puromycin (1 μg/mL).

## References

[1] Ahn BY, Trinh DL, Zajchowski LD, Lee B, Elwi AN, Kim SW (2010). Tid1 is a new regulator of p53 mitochondrial translocation and apoptosis in cancer. Oncogene.

[2] Fuster JJ, Sanz-Gonzalez SM, Moll UM, Andres V (2007). Classic and novel roles of p53: prospects for anticancer therapy. Trends Mol Med.

[3] Koumenis C, Alarcon R, Hammond E, Sutphin P, Hoffman W, Murphy M, Derr J, Taya Y, Lowe SW, Kastan M, Giaccia A (2001). Regulation of p53 by hypoxia: dissociation of transcriptional repression and apoptosis from p53-dependent transactivation. Mol Cell Biol.

[4] Lohr K, Moritz C, Contente A, Dobbelstein M (2003). p21/CDKN1A mediates negative regulation of transcription by p53. J Biol Chem.

[5] Riley T, Sontag E, Chen P, Levine A (2008). Transcriptional control of human p53-regulated genes. Nat Rev Mol Cell Biol.

[6] Slee EA, O'Connor DJ, Lu X (2004). To die or not to die: how does p53 decide?. Oncogene.

[7] Moll UM, Zaika A (2001). Nuclear and mitochondrial apoptotic pathways of p53. FEBS Lett.

[8] Moll UM, Wolff S, Speidel D, Deppert W (2005). Transcription-independent pro-apoptotic functions of p53. Curr Opin Cell Biol.

[9] Marchenko ND, Zaika A, Moll UM (2000). Death signal-induced localization of p53 protein to mitochondria. A potential role in apoptotic signaling.. J Biol Chem.

[10] Erster S, Moll UM (2005). Stress-induced p53 runs a transcription-independent death program. Biochem Biophys Res Commun.

[11] Erster S, Moll UM (2004). Stress-induced p53 runs a direct mitochondrial death program: its role in physiologic and pathophysiologic stress responses in vivo. Cell Cycle.

[12] Talos F, Petrenko O, Mena P, Moll UM (2005). Mitochondrially targeted p53 has tumor suppressor activities in vivo. Cancer Res.

[13] Genevaux P, Georgopoulos C, Kelley WL (2007). The Hsp70 chaperone machines of Escherichia coli: a paradigm for the repartition of chaperone functions. Mol Microbiol.

[14] Hafizur RM, Yano M, Gotoh T, Mori M, Terada K (2004). Modulation of chaperone activities of Hsp70 and Hsp70-2 by a mammalian DnaJ/Hsp40 homolog, DjA4. J Biochem.

[15] Mitra A, Shevde LA, Samant RS (2009). Multi-faceted role of HSP40 in cancer. Clin Exp Metastasis.

[16] Qiu XB, Shao YM, Miao S, Wang L (2006). The diversity of the DnaJ/Hsp40 family, the crucial partners for Hsp70 chaperones. Cell Mol Life Sci.

[17] Walsh P, Bursac D, Law YC, Cyr D, Lithgow T (2004). The J-protein family: modulating protein assembly, disassembly and translocation. EMBO Rep.

[18] Cyr DM, Langer T, Douglas MG (1994). DnaJ-like proteins: molecular chaperones and specific regulators of Hsp70. Trends Biochem Sci.

[19] Lu S, Van EJ, Zhou X, Lopez AB, O'Halloran DM, Cosman KM, Conlin BJ, Paolillo DJ, Garvin DF, Vrebalov J, Kochian LV, Kupper H, Earle ED, Cao J, Li L (2006). The cauliflower Or gene encodes a DnaJ cysteine-rich domain-containing protein that mediates high levels of beta-carotene accumulation. Plant Cell.

[20] Cheng H, Cenciarelli C, Tao M, Parks WP, Cheng-Mayer C (2002). HTLV-1 Tax-associated hTid-1, a human DnaJ protein, is a repressor of Ikappa B kinase beta subunit. J Biol Chem.

[21] Kim SW, Chao TH, Xiang R, Lo JF, Campbell MJ, Fearns C, Lee JD (2004). Tid1, the human homologue of a Drosophila tumor suppressor, reduces the malignant activity of ErbB-2 in carcinoma cells. Cancer Res.

[22] Liu HY, MacDonald JI, Hryciw T, Li C, Meakin SO (2005). Human tumorous imaginal disc 1 (TID1) associates with Trk receptor tyrosine kinases and regulates neurite outgrowth in nnr5-TrkA cells. J Biol Chem.

[23] Trentin GA, Yin X, Tahir S, Lhotak S, Farhang-Fallah J, Li Y, Rozakis-Adcock M (2001). A mouse homologue of the Drosophila tumor suppressor l(2)tid gene defines a novel Ras GTPase-activating protein (RasGAP)-binding protein. J Biol Chem.

[24] Syken J, De-Medina T, Munger K (1999). TID1, a human homolog of the Drosophila tumor suppressor l(2)tid, encodes two mitochondrial modulators of apoptosis with opposing functions. Proc Natl Acad Sci U S A.

[25] Wadhwa R, Yaguchi T, Hasan MK, Mitsui Y, Reddel RR, Kaul SC (2002). Hsp70 family member, mot-2/mthsp70/GRP75, binds to the cytoplasmic sequestration domain of the p53 protein. Exp Cell Res.

[26] Kaul SC, Aida S, Yaguchi T, Kaur K, Wadhwa R (2005). Activation of wild type p53 function by its mortalin-binding, cytoplasmically localizing carboxyl terminus peptides. J Biol Chem.

[27] Endo T, Mitsui S, Roise D (1995). Mitochondrial presequences can induce aggregation of unfolded proteins. FEBS Lett.

[28] Sugito K, Yamane M, Hattori H, Hayashi Y, Tohnai I, Ueda M, Tsuchida N, Ohtsuka K (1995). Interaction between hsp70 and hsp40, eukaryotic homologues of DnaK and DnaJ, in human cells expressing mutant-type p53. FEBS Lett.

[29] King FW, Wawrzynow A, Hohfeld J, Zylicz M (2001). Co-chaperones Bag-1, Hop and Hsp40 regulate Hsc70 and Hsp90 interactions with wild-type or mutant p53. EMBO J.

